# Breg-Mediated Immunoregulation in the Skin

**DOI:** 10.3390/ijms25010583

**Published:** 2024-01-02

**Authors:** Elina A. Zheremyan, Alina S. Ustiugova, Nina M. Karamushka, Aksinya N. Uvarova, Ekaterina M. Stasevich, Apollinariya V. Bogolyubova, Dmitry V. Kuprash, Kirill V. Korneev

**Affiliations:** 1 Center for Precision Genome Editing and Genetic Technologies for Biomedicine, Engelhardt Institute of Molecular Biology, Russian Academy of Sciences, 119991 Moscow, Russia; 2 Faculty of Biology, Lomonosov Moscow State University, 119234 Moscow, Russia; 3 National Research Center for Hematology, 125167 Moscow, Russia

**Keywords:** regulatory B cells, skin homeostasis, wound healing, inflammatory skin pathology, tissue regeneration, immune regulation

## Abstract

Wound healing is a complex process involving a coordinated series of events aimed at restoring tissue integrity and function. Regulatory B cells (Bregs) are a subset of B lymphocytes that play an essential role in fine-tuning immune responses and maintaining immune homeostasis. Recent studies have suggested that Bregs are important players in cutaneous immunity. This review summarizes the current understanding of the role of Bregs in skin immunity in health and pathology, such as diabetes, psoriasis, systemic sclerosis, cutaneous lupus erythematosus, cutaneous hypersensitivity, pemphigus, and dermatomyositis. We discuss the mechanisms by which Bregs maintain tissue homeostasis in the wound microenvironment through the promotion of angiogenesis, suppression of effector cells, and induction of regulatory immune cells. We also mention the potential clinical applications of Bregs in promoting wound healing, such as the use of adoptive Breg transfer.

## 1. Introduction

Inflammatory diseases are often accompanied by cutaneous abnormalities, ranging from minor skin irritation to severe chronic conditions [1]. In such diseases, the inflammatory process of the skin is usually driven by the activation of the immune system and the production of inflammatory mediators [2]. These inflammatory mediators cause vasodilation and increase vascular permeability, allowing immune cells to migrate to the site of inflammation. The activated immune cells then release additional pro-inflammatory cytokines and chemokines, amplifying the immune response and leading to further tissue damage and inflammation. Cutaneous wound healing is a complex process aimed at restoring skin tissue homeostasis and preventing further damage and infection. This process unfolds in an intricate sequence of phases, often occurring simultaneously rather than in a strictly linear fashion [3]. The initial inflammatory phase, encompassing vascular and cellular responses, commences with hemostasis marked by platelet aggregation and fibrin clot formation [4]. Subsequently, immune cells such as neutrophils, monocytes and lymphocytes infiltrate the wound bed, undertaking crucial roles in tissue debridement and cytokine release [5]. Transitioning to the repair phase involves the creation of granulation tissue, re-epithelialization and wound contraction, with fibroblasts, endothelial cells and keratinocytes as key players. Fibroblasts contribute to matrix formation, while endothelial cells drive angiogenesis. Keratinocytes migrate to cover the wound and myofibroblasts aid in contraction [6]. The maturation phase focuses on tissue remodeling, apoptosis and collagen cross-linking, leading to the formation of an acellular scar [7]. Thus, the complex process of wound healing is orchestrated by multiple immune cell types [8,9,10,11,12]. However, the role of B cells, critical players in the systemic immune response, still needs to be fully understood in the context of the local immune response in the skin. One type of B cell that deserves attention in the study of the wound healing processes is regulatory B cells (Bregs). The count of diverse subpopulations of regulatory B cells currently exceed ten [13] and unfortunately, there is no universally accepted specific marker that sets them apart from other B cell subsets. As a result, the term “Breg” is commonly used as a general label, denoting B cells with immunosuppressive potential [14,15,16]. Bregs suppress immune responses and promote tissue repair by producing anti-inflammatory cytokines and interacting with other immune cells [17]. Almost 50 years ago, it was discovered that regulatory B cells may play a role in supporting the healing process of cutaneous wounds [18]. Despite this finding, research into the role of Bregs in wound healing has remained limited, possibly due to the challenge of identifying these cells with clear phenotypic markers. Since Bregs are a relatively rare and diverse population of B lymphocytes, it can be challenging to distinguish them from other B cell subsets [19].

In general, Bregs are known to exhibit a distinct range of immunosuppressive mechanisms, which encompass both soluble factors (IL10, IL35, TGFβ, granzyme B, etc.) and surface molecules (PD-L1, FasL, CD39, CD73, etc.) [20,21,22,23,24,25,26]. Several novel molecules (ADORA2B, NID1, CysF, SLAMF7, etc.) involved in Breg-mediated immunosuppression have also been proposed as a result of transcriptome analysis [27]. Despite such a rich diversity of molecular mechanisms, most studies of skin Bregs have focused on the subpopulation of IL10-producing cells. Previous studies have shown that imiquimod-induced psoriasis-like skin inflammation in IL10-deficient mice was more severe and persistent than in wild-type mice [28]. The importance of IL10 as a scar modulator has also been demonstrated in IL10 knock-out (KO) mouse embryos. Cutaneous injury in these KO mice resulted in adult-like scar-mediated healing that was not observed in wild-type embryonic counterparts [29].

Within this emerging area of B cell immunology, persistent questions surround the migration and maintenance of regulatory B cells in the skin, along with the identification of specific molecular signatures defining their regulatory roles in cutaneous immunity. The significance of various Breg subsets in sustaining cutaneous immunity adds further complexity to the landscape. The cumulative evidence points toward a constructive role of Bregs in the realm of wound healing and the suppression of inflammation across diverse cutaneous pathologies. This insight opens the door to potential therapeutic avenues, with regulatory B cell immunotherapy emerging as a promising strategy to not only foster wound healing but also to address the challenges posed by chronic inflammatory and autoimmune skin disorders. In this review, we discuss the variety of potential mechanisms of Breg-mediated wound healing: maintenance of tissue homeostasis, promotion of angiogenesis, suppression of effector cells, and induction of regulatory immune cells, and highlight the role of Bregs in inflammatory-associated skin pathologies.

## 2. Breg Homing and Induction in the Skin

Traditionally, B cells were thought to accumulate in the skin during inflammation but not under homeostatic conditions. However, B cells were later identified and characterized in the skin of humans, mice, and sheep, even in the absence of inflammation [30,31]. Within mammalian skin, B cells primarily reside in the dermis, exhibiting sparse dispersion as individual cells in homeostatic conditions and forming cell clusters or organized lymphoid structures during inflammation [17]. Based on the current limited knowledge, the homing and migration mechanisms of Bregs do not appear to be dramatically different from those of other B cells. In the classical paradigm of lymphocyte trafficking, orchestrated migration is governed by interactions involving distinct combinations of tissue-specific adhesion molecules (such as selectins and integrins) and their interplay with chemokines and corresponding receptors. This complex interplay ultimately facilitates the migration of cells through vascular endothelia and their infiltration into tissue sites [32]. In particular, the expression of α4β1 integrin on B cells has been identified as a critical factor for Breg homing to tissues, including skin [33]. The CCL20–CCR6 axis has also been shown to be essential for B cell migration from lymph to skin [31]. Another stimulus that leads to Breg induction in the skin is moderate ultraviolet exposure, which has been shown to activate resident Bregs in the skin that can inhibit dendritic cell activation of T cells in an IL10-dependent manner in models of cutaneous hypersensitivity [17,34].

## 3. Bregs Promote Cutaneous Wound Healing via Several Mechanisms in a Variety of Pathological and Healthy States

**Figure 1:**
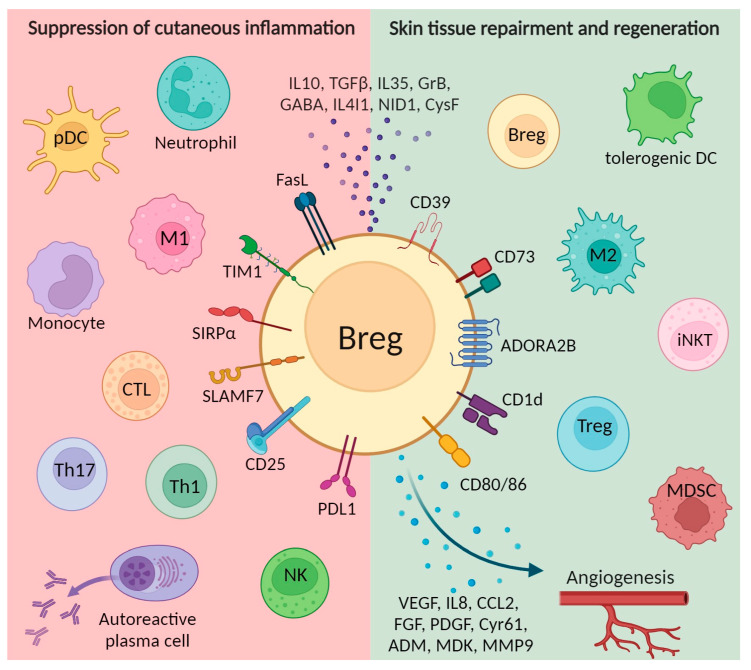
Immunosuppressive mechanisms involved in Breg-mediated cutaneous wound healing include suppression of inflammatory responses. Mechanisms involved in inhibiting effector cells are shown in the red area on the left; activation of suppressor cell types and promotion of angiogenesis are depicted in the green area on the right. Breg, regulatory B cells; CTL, cytotoxic T cells; iNKT, invariant natural killer T cells; M1, classically activated macrophages; M2, alternatively activated macrophages; MDSC, myeloid-derived suppressor cells; NK, natural killers; pDC, plasmacytoid dendritic cells; Th, T helper cells; Treg, regulatory T cells. Created with BioRender.com, accessed on 24 November 2023.

In this section, we discuss how Bregs contribute to the maintenance of tissue homeostasis in the wound microenvironment by fostering angiogenesis, suppressing effector cells, and prompting the generation of regulatory immune cells. The summarized depiction of the below-mentioned mechanisms can be found in Figure 1.

### 3.1. Maintaining Tissue Homeostasis

Anti-inflammatory cytokines, such as IL10 and TGFβ, are known to influence both immune cells and epithelial cells during wound healing [35,36]. Interestingly, the isoforms of TGFβ have different roles in wound healing machinery. In comparison, TGFβ1 and TGFβ2 are key players in the proliferative phase of wound healing, where they serve to promote signaling via SMAD and Wnt-dependent pathways to enhance scarring, whereas TGFβ3 reduces scarring [29]. Geherin and colleagues showed that Bregs reside in murine cutaneous tissue, where they accumulate during inflammation and secrete IL10. The authors also found that human IL10-producing B cells (B10) in the skin are similar to their mouse counterparts indicating that human B10 cells are a part of the normal skin immune system [30]. In mouse models, IL10^+^ Bregs reduced inflammation in several pathologies, including cutaneous hypersensitivity, scleroderma, and psoriasis-like inflammation [37,38,39]. Patients with psoriasis and other cutaneous inflammatory diseases have reduced numbers of circulating IL10^+^ Bregs during active disease [17]. In vivo experiments on mice showed that up to 30% of B cells applied onto the skin wound acquired an immunomodulatory phenotype after such adoptive cell transfer. B cell treatment reduced the expression of TNF and increased IL10 and TGFβ at the injury site. In addition, B cells reduced the expression of inflammatory molecules and increased proteins associated with proliferation, tissue remodeling, and protection from oxidative stress [40]. Regarding the molecular mechanisms underlying these processes, the authors showed that MyD88-dependent toll-like receptor (TLR) signaling through TLR2/6 and TLR4 is essential for the protective benefit of B cells in repairing injured tissues in vivo [40].

### 3.2. Promotion of Angiogenesis

Angiogenesis is an essential physiological process occurring during repair after cutaneous injury [41]. Pathological angiogenesis has also been observed in carcinogenesis and chronic inflammation [42]. Van de Veen and colleagues identified a subpopulation of CD49b^+^CD73^+^ Bregs with high angiogenic potential that produces various angiogenic factors, including vascular endothelial growth factor (VEGF), fibroblast growth factor (FGF), platelet-derived growth factor (PDGF), hepatocyte growth factor, axon guidance factor, and angiopoietins, orchestrating the formation of endothelial cell tubes [43]. Other Breg-produced factors, including cysteine-rich angiogenic inducer 61 (CYR61), adrenomedullin (ADM), and midkine (MDK), have also been reported to promote angiogenesis [44,45,46]. Free adenosine (which can be generated by the ectoenzymes CD39 and CD73, which are highly expressed on Bregs) also promotes angiogenesis through its direct mitogenic effects on endothelial cells and induction of proangiogenic factors (VEGF, IL8, FGF) from vascular and immune cells [47].

In patients with melanoma, B cells express several angiogenic factors, such as sphingosine-1-phosphate receptor 1 (S1PR1), matrix metallopeptidase 9 (MMP9), hypoxia-inducible factor 1-alpha (HIF1a), and VEGF. B cells were also found to accumulate around vessels rather than spread throughout the tissue; these B lymphocytes exhibited persistently phosphorylated STAT3, which appeared to be required for pro-angiogenic gene expression [48].

Further evidence for the role of Bregs in the promotion of angiogenesis was provided by Zhang et al. CD5^+^ Bregs were found to be activated by direct ligation of CD5 by IL6 (in the absence of IL6R), leading to the upregulation of STAT3. These processes led to the secretion of the pro-angiogenic factors VEGF and CCL2 by Bregs [49].

### 3.3. Interaction with Other Types of Suppressor Cells

#### 3.3.1. Regulatory T Cells

Regulatory T cells (Tregs) help to suppress excessive immune responses and promote immune tolerance. Tregs are important for regulating the inflammatory response in wound healing to prevent excessive tissue damage and promote tissue repair [50]. The skin regulatory T cell population comprises both resident and circulating Tregs, which can accumulate in the injured tissue [4]. Haertel and colleagues demonstrated that Treg depletion compromised normal acute wound healing: impaired re-epithelization, reduced wound closure, and delayed angiogenesis [51]. B cells play a crucial role in promoting the differentiation and activation of Tregs, as demonstrated by the reduced frequency of Foxp3^+^ Tregs in B cell-deficient mice [52]. Human CD19^+^CD25^hi^ Bregs were found to suppress the proliferation of CD4^+^ T cells and increase Foxp3 and CTLA4 expression in Tregs via direct contact (CD86, CD1d) and TGFβ secretion [53]. Another group demonstrated that naive B cells can facilitate the differentiation of naive T cells into Foxp3^−^ regulatory T cells in an antigen-specific manner [54]. Chien et al. showed that naive B cells can convert CD4^+^CD25^−^ T cells into CD25^+^Foxp3^−^ regulatory T cells that express a variety of negative costimulatory receptors and secrete IL10 [55]. Bregs can also generate adenosine, which acts on the adenosine A2A receptor on T cells and promotes their differentiation into Tregs [56]. Bregs are also capable of producing the secreted L-amino acid oxidase IL4I1 during inflammatory responses, which can lead to H_2_O_2_-dependent suppression of effector cell functions and generation of Tregs and alternatively activated macrophages as well [57].

#### 3.3.2. Alternatively Activated (M2) Macrophages

Macrophages have critical functions at each stage of wound healing, namely inflammation, proliferation, and remodeling. Throughout the healing process, macrophages shift from primarily exhibiting pro-inflammatory traits (M1-like phenotypes) to displaying anti-inflammatory features (M2) [58]. However, chronic wounds that do not heal, such as diabetic ulcers, remain in the inflammatory phase of wound healing, and as a result, macrophages in the wound area retain pro-inflammatory properties [59]. Bregs can shift macrophage polarization from M1 to M2 by producing anti-inflammatory cytokines (IL10, TGFβ, etc.). For instance, IL10^+^CD1d^+^CD5^+^ Bregs are known to polarize M2 macrophages, thereby limiting damaging responses [60]. The newly discovered Breg-derived γ-aminobutyric acid (GABA) was also found to induce monocytes to differentiate into anti-inflammatory macrophages [61]. Moreover, Bregs can decrease CCR2-mediated monocyte recruitment and mobilization, thus preventing the further promotion of inflammatory processes [62].

#### 3.3.3. Other Immune Suppressor Cells

Invariant natural killer T cells (iNKT) promote skin wound healing by preventing a prolonged neutrophilic inflammatory response [63]. Bregs support iNKT proliferation, activation, and cytokine production in a CD1d-dependent manner [20]. Bregs can also modulate dendritic cell (DC) function in inflammation by reducing antigen presentation and pro-inflammatory cytokine productions by DCs or by modulating monocytes to differentiate into tolerogenic DCs [20].

Overall, these data suggest that Bregs possess multiple mechanisms to influence the differentiation, recruitment, and activation of other types of suppressor cells. By limiting excessive inflammation and promoting tissue repair, Bregs and other suppressor cells may work together to ensure proper wound healing and prevent the development of chronic wounds or other cutaneous complications.

### 3.4. Suppression of Effector Cells

#### 3.4.1. Cytotoxic T Cells

Although the main immunosuppressive effect of Bregs on T cells is believed to be Treg induction, as discussed above, there are other mechanisms worth mentioning. Cytotoxic T cells are active participants in the early stages of acute wound healing [5]. Bregs suppressed the production of CCL5—a potent skin chemoattractant—by the skin epidermis preventing the infiltration of effector CD8^+^ T cells into the skin [64]. IL10-producing B cells have been described to suppress IFNγ and GrB expression by T killers [65,66]. Breg-secreted TGFβ increased reactive oxygen species (ROS) and NO production by myeloid-derived suppressor cells (MDSC), which inhibited cytotoxic T cell proliferation [67]. TGFβ can also induce T killer cell anergy [68]. Bregs can also suppress CD8^+^ T cell activity via the PD-1–PD-L1 inhibitory pathway [69].

#### 3.4.2. Helper T Cells

Regarding helper T cells (Th), it has been reported that Bregs can suppress both Th1 and Th17 responses [70]. This process may be very beneficial in the case of diabetic ulcers, since the level of these inflammatory T helper subsets is increased in patients with diabetes mellitus [71]. B cell-derived TGFβ was found to inhibit Th1 and Th17 immune responses by decreasing the antigen-presenting capacity of dendritic cells [72]. B10 cells also inhibit Th1 and Th17 cells through IL10 production, thus ameliorating experimental arthritis [73]. Bregs have been found to induce CD4^+^ T cell death in general: experiments in diabetes models revealed that LPS-stimulated B cells express FasL and TGFβ and can induce apoptosis of both effector B and T lymphocytes [74].

#### 3.4.3. Natural Killer Cells

During the late phases of wound healing, natural killer (NK) cells have a predominantly negative impact on tissue repair. NK-produced IFNγ polarizes macrophages toward the pro-inflammatory M1 phenotype, thereby enhancing immune cell infiltration at the wound site via macrophages producing cytokines such as IL1β, IL6, IL12, IL23, and TNF [5,75]. Therefore, NK-mediated inflammatory processes may hinder wound healing, especially in chronic wounds. In the immunosuppressive tumor setting, Bregs have been reported to suppress IFNγ production by NK cells via the production of IL10 [76] and to abrogate NK cell-mediated cytotoxicity [77,78]. Transcriptome analysis suggests that Bregs may produce several proteins with suppressive effects on NK cell activating receptors and effector mechanisms [27].

#### 3.4.4. Dendritic Cells

The presence of plasmacytoid dendritic cells (pDCs) has been detected in cutaneous healing processes [79]. A regulatory feedback loop between pDCs and B cells has been demonstrated in both humans and mice. In response to inflammation, pDCs secrete IFNα, which induces B cells to produce IL10, subsequently suppressing IFNα production by pDCs. This regulatory feedback can be disrupted in autoimmune conditions such as systemic lupus erythematosus [80]. IL10^+^ B cells were found to inhibit dendritic cell function to generate pathogenic T cells [81].

#### 3.4.5. Neutrophils

Neutrophils are among the first circulating immune cells recruited to a wound that contribute to tissue injury by amplifying the inflammatory response [82]. The ability of Bregs to suppress neutrophils plays an important role in the proper resolution of an inflammatory response as shown in murine models of colitis and salmonella infection [83,84]. Peripheral IgM^+^ transitional B cells with regulatory properties were found to dampen excessive neutrophil activity in the lung [85].

#### 3.4.6. Effector B Cells

Effector B cells can also be suppressed by their regulatory counterparts. Rosser and colleagues proposed three possible mechanisms for Breg-mediated suppression of antibody responses: (1) direct suppression of antibody-producing B cells; (2) suppression of T helper responses, which will inevitably lead to a decrease in plasma cell generation; (3) induction of Tregs, which could contribute to global suppression of inflammatory responses, including suppression of antibody production [70]. However, except for pathogenic plasma cells, there are also regulatory plasma cells which have been recently identified. IgM^+^CD19^+^CD138^hi^ plasma cells were shown to produce anti-inflammatory cytokines (IL10, IL35) during autoimmune and infectious conditions, thus suggesting their regulatory role in inflammation resolution [86].

Thus, these data allow us to delve into the intricate mechanisms through which regulatory B cells exert their suppressive influence on a range of effector cells. From inhibiting the infiltration of CD8^+^ T cells into the skin by modulating chemoattractant production to suppressing various T cell responses and orchestrating an immunosuppressive environment, Bregs emerge as crucial regulators in immune homeostasis. The multifaceted impact extends to NK cells, dendritic cells, and neutrophils, contributing to the resolution of inflammatory responses and tissue repair. Additionally, the regulatory interplay between Bregs and effector B cells highlights the broader implications for antibody responses. As we unravel the complexity of these interactions, the potential therapeutic applications of harnessing Breg-mediated immunosuppression become increasingly apparent, holding promise for addressing diverse pathological conditions, including chronic wounds, autoimmune disorders, and inflammatory responses.

## 4. Breg-Mediated Wound Healing in Inflammatory Skin Diseases

**Figure 2:**
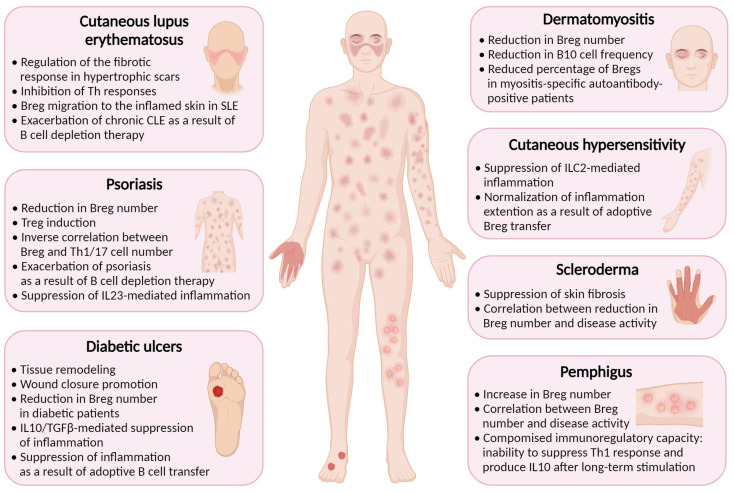
Regulatory B cells are implicated in the pathogenesis of inflammatory skin diseases. Created with BioRender.com, accessed on 24 November 2023.

Inflammation is a natural response of the immune system to injury or infection, and it plays a vital role in the healing process. Bregs have been found to play a role in wound healing and suppression of inflammation in several cutaneous pathologies associated with skin damage. Some inflammatory diseases, such as psoriasis, systemic sclerosis (scleroderma), cutaneous lupus erythematosus, cutaneous hypersensitivity, pemphigus, dermatomyositis, and diabetes, have been found to be associated with reduced Breg function (Figure 2) [17].

### 4.1. Diabetes Mellitus

Diabetes is a chronic metabolic disorder that impairs the body’s ability to regulate blood glucose levels. In diabetes, chronic hyperglycemia, and impaired immune function, among other complications, can lead to a persistent state of low-grade inflammation with excessive production of cytokines and chemokines exacerbating skin ulceration and impairing the healing process [87,88]. Bregs are decreased in patients with diabetes [89], possibly contributing to delayed wound healing. Adoptive transfer of mature naive B cells to diabetic ulcers in mice resulted in increased cutaneous levels of homeostatic (IL6) and anti-inflammatory (IL10, TGFβ) cytokines contributing to tissue remodeling, reduced inflammation, and wound closure [19,90].

### 4.2. Psoriasis

Psoriasis is a chronic autoimmune disease affecting skin, nails, and joints [91]. In individuals with psoriasis, the immune system responds abnormally to skin cells, causing them to grow rapidly and resulting in a buildup of cells on the surface of the skin [92]. Patients with psoriasis have lower levels of IL10^+^ B cells in their bloodstream, which is consistent with studies in mice showing the crucial role of these cells in suppressing psoriasiform inflammation [37,93]. A number of IL10^+^ Breg cells was found to be inversely correlated with psoriasis severity and the number of pathogenic IL17A^+^CD3^+^ and IFNγ^+^CD3^+^ T cells [94]. In fact, restricted migration of Bregs into the skin of mice with B cell-specific disruption of α4β1 integrin expression resulted in a delayed resolution of psoriasiform skin inflammation [33]. Bregs also suppressed IL23-mediated psoriatic inflammation through Treg expansion and inhibition of Th17 responses [95]. Conversely, the elimination of beneficial Bregs may explain why some B cell-depleting therapies were found to exacerbate psoriasis [96].

### 4.3. Contact Hypersensitivity

Contact hypersensitivity (CHS) is an immune-mediated skin reaction that clinically manifests as allergic contact dermatitis [97]. Both CD4^+^ and CD8^+^ T cells have been shown to play a pivotal role in the progression of CHS [98]. The immune response is accompanied by the release of cytokines and other immune mediators that cause inflammation and the characteristic symptoms of CHS, such as redness, itching, and blistering. An enhanced CHS response in CD19-deficient mice was attenuated by the transfer of wild-type Bregs, indicating that CD19 expression may be necessary for Breg differentiation and function in this context [99]. IL10^+^ Breg cells were found to suppress the activation of IL13-producing type 2 innate lymphoid cells in mice with oxazolone-induced severe CHS [100], further highlighting the potential role of Bregs in regulating immune responses and promoting wound healing [101].

### 4.4. Systemic Sclerosis (Scleroderma)

Systemic sclerosis is a chronic autoimmune disease that affects the skin and internal organs. It is characterized by the excessive production and deposition of collagen in various body tissues, leading to the hardening and thickening of the skin and other tissues [102]. Systemic sclerosis can vary widely in its severity and manifestations, and conservative treatment options rely upon immunosuppressive medications to reduce the immune response and alleviate symptoms [103]. Studies have shown that IL10-producing Bregs can suppress skin fibrosis in the Scl-cGVHD (sclerodermatous chronic graft-versus-host disease) model, an animal model of human systemic sclerosis [104]. Patients with systemic sclerosis also demonstrate a reduction in IL10^+^ Bregs associated with disease activity [38,105]. These data suggest that Bregs play a beneficial role in the complex role of B cells in systemic sclerosis [106].

### 4.5. Cutaneous Lupus Erythematosus

Cutaneous lupus erythematosus (CLE) is a cutaneous manifestation of systemic lupus erythematosus (SLE) that affects the skin, occurring in 80% of cases [107]. In a mouse model of SLE, induction of Bregs has been found to suppress both skin and systemic disease [108]. In SLE patients, IL10^+^ B cells localize in the inflamed skin [109]. Patients with hypertrophic scars demonstrate a higher percentage of Breg cells compared to healthy donors, suggesting that Bregs may regulate the fibrotic response in CLE, presenting a potential therapeutic target [110]. A study in SLE patients showed that the CD19^+^CD24^hi^CD38^hi^ B cell subset produced less IL10 in response to CD40 stimulation and was unable to inhibit Th responses, suggesting a functional impairment of Bregs [111]. Evaluation of the clinical response to B cell depletion therapy with rituximab showed that none of the patients with chronic CLE responded, and new lesions were even observed [112]. One of the possible reasons for this could be the depletion of regulatory B cells, which promote immunosuppression of the inflammatory response in chronic CLE. These findings support the potential beneficial role of Bregs in chronic CLE.

### 4.6. Pemphigus

Pemphigus is a rare autoimmune disease that is characterized by intraepidermal blisters caused by autoantibodies against desmoglein 1 and 3 [113]. One study has revealed that the frequency of CD19^+^CD24^hi^CD38^hi^ Bregs in PBMCs of pemphigus patients was elevated compared with healthy controls, but they were functionally impaired, unable to suppress Th1 immune response [113]. The increase in Breg number can be linked with elevated IL21 level, which is characteristic of pemphigus patients, and is known to promote Bregs [114]. Moreover, Breg ratio was found to be dependent on the disease activity: their frequency in patients with active disease compared with that in the remittent patients. Bregs isolated from PBMCs of patients with pemphigus secreted normal levels of IL10 after ex vivo stimulation but showed compromised capacity of producing IL10 after long-term stimulation, which can occur in vivo during disease progression [115]. In a study on rituximab-treated pemphigus patients, Bregs restored their IL10-secreting capacity in patients with complete remission, but not in no response and incomplete remission, thus supporting the idea of a significant role of B10 cells in pemphigus pathogenesis [116].

### 4.7. Dermatomyositis

Dermatomyositis (DM) is a systemic autoimmune disease primarily affecting skin and muscles and which is characterized by dysregulation of hyperactivated B and T cells, leading to autoantibody production and aberrant cytokine production [117]. In DM patients, aggregates of lymphocytes are found in skin lesions [118]. A very comprehensive study conducted by Li et al. revealed that Breg deficiency is an important immunopathogenic feature of DM [119]. CD19^+^CD24^hi^CD38^hi^ Breg level was found to be significantly decreased in the peripheral blood of DM patients, and IL10^+^ B cell levels were also lower compared to those of healthy controls. Myositis-specific autoantibody (MSA)-positive patients were shown to have lower percentage of CD19^+^CD24^hi^CD38^hi^ Bregs than in MSA-negative individuals, thus supporting possible role of Bregs in the control of autoantibody production [119].

Taken together, the data presented underscores the pivotal role of regulatory B cells in various cutaneous pathologies. From diabetes, psoriasis, and contact hypersensitivity to systemic sclerosis, cutaneous lupus erythematosus, pemphigus, and dermatomyositis, Bregs demonstrate a consistent and beneficial impact on immune responses and wound healing. Their ability to suppress inflammation, modulate cytokine production, and influence the severity of autoimmune conditions highlights the therapeutic potential of harnessing Breg-mediated immunosuppression. Recognizing the nuanced interactions between Bregs and different immune cells provides valuable insights for developing targeted strategies to optimize immune responses and enhance healing processes in diverse inflammatory skin disorders.

## 5. Conclusions

There has been a growing interest in studying the role of the immune system in cutaneous tissue repair and regeneration. However, the precise mechanisms by which the adaptive immune system regulates wound healing remain poorly understood. As we have tried to demonstrate in our synthesis, a number of recent studies have shown the importance of Bregs in resolving inflammation and promoting wound healing, making further study of Bregs in skin tissue repair a promising direction. Understanding Breg skin homing mechanisms could facilitate the development of targeted therapies to manipulate Breg trafficking and functioning. Additionally, the identification of specific Breg subsets that are involved could lead to the development of novel cell therapy approaches to improve skin homeostasis. Over the past few decades, therapies specifically targeting B cells have proven highly effective in the clinical realm, particularly in the management of autoimmune diseases [120,121]. At the same time, the diverse range of immunosuppressive mechanisms exhibited by Bregs has positioned the adoptive transfer of these regulatory B cells as a promising therapeutic strategy for conditions marked by the overactivation of immune responses [78,122]. Looking forward, the potential of Breg cell therapy emerges as a beacon of hope for the treatment of cutaneous wounds, including the formidable challenge of chronic wounds resistant to current approaches.

## Data Availability

Not applicable.
